# Job stress, a source of hypertension among workers in Sub-Saharan Africa: a scoping review

**DOI:** 10.1186/s12889-023-17248-5

**Published:** 2023-11-23

**Authors:** Rodrigue Khonde Kumbu, Hervé Matondo, Aline Labat, Bernard Kianu, Isabelle Godin, Guillaume Kiyombo, Yves Coppieters

**Affiliations:** 1 Environmental Health, Kinshasa School of Public Health, University of Kinshasa, Kinshasa, Democratic Republic of Congo; 2 Biostatistics and Clinical Research, School of Public Health, Research Centre in Epidemiology, Université Libre de Bruxelles (ULB), Brussels, Belgium; 3 Unit of Cardiology, University Clinic of Kinshasa, University of Kinshasa, Kinshasa, Democratic Republic of Congo; 4 Research Centre in Social Approaches to Health, School of Public Health, Université Libre de Bruxelles (ULB), Brussels, Belgium

**Keywords:** Hypertension, Job stress, Workers, Sub-Saharan Africa, Scoping review

## Abstract

**Background:**

Hypertension remains one of the leading risk factors for cardiovascular disease. Contrasting with the high-income countries where the rates of hypertension decline, it increases in Sub-Saharan African countries. The age group most affected by hypertension is the working population. Several studies carried out in Europe, North America, and Asia, underline the influence of job stress on the occurrence of hypertension. The objective of this review was to explore current knowledge about hypertension and job stress in Sub-Saharan Africa.

**Methods:**

We conducted a scoping review using Arksey and O’Malley’s framework to synthesize findings. We searched in PubMed, Scopus, and ProQuest databases. The inclusion criteria were peer-reviewed manuscripts published on March 1, 2023, conducted among workers in Sub-Saharan Africa, reported hypertension and job stress, and using quantitative methodologies. Data were assessed independently by two researchers.

**Results:**

In total, 295 articles were identified from databases. Of these, only 12 articles met the inclusion criteria and were included in the review (9 cross-sectional studies and 3 case–control studies). These studies focused on sectors reported as stressful (health, banking, education, and industries). The prevalence of hypertension varied from 14.3% to 45.9%, with a high proportion of hypertensive participants (35.4%-70.6%) who were unaware that they had hypertension. Job stress was significantly associated with hypertension (OR = 2.4 [1.5–4.4]) and stress management was inversely associated with hypertension (*r* = -0.14, *p* < 0.05). However, no study reported an existing workplace health promotion program implemented, especially regarding cardiovascular disease risk factors.

**Conclusion:**

Data available in the literature show that stressful working conditions may be associated with hypertension. We are faced with an increasing prevalence of hypertension among workers in Sub-Saharan Africa, where a large proportion of them are unaware that they have hypertension. Thus, there is a need to implement workplace prevention and health promotion strategies in Sub-Saharan Africa.

**Supplementary Information:**

The online version contains supplementary material available at 10.1186/s12889-023-17248-5.

## Background

Worldwide, hypertension (HTN) or high blood pressure remains one of the leading preventable risk factors for disease and death if not detected early and treated adequately [1]. HTN is the primary modifiable risk factor for cardiovascular disease, impacting over one billion individuals worldwide, and contributing to over 10 million avoidable premature deaths annually [2, 3]. WHO estimates that 1.28 billion adults aged 30–79 are affected. Of these, two-thirds are estimated to live in low and middle-income countries, with 27% in the WHO African Region [4]. In contrast to the high-income countries where the rates of HTN decline, it is increasing in sub-Saharan African countries [5–7].

HTN is defined by the JNC8 Panel Member Report in the general population as systolic blood pressure (SBP) ⩾ 140 mmHg and in individuals aged 60 years and older without diabetes or chronic kidney disease by SBP ⩾150 mmHg and by diastolic blood pressure (DBP) ⩾90 mmHg [8]. The etiology of HTN is complex and multifactorial. The genetic and behavioral factors known to date to be involved in the genesis of HTN explain in part the variability of results observed. A large number of studies have looked at psychosocial stress as another possible risk factor [9]. Recent data highlight the important role of nontraditional risk factors in the development of HTN, such as psychosocial stressors including job stress [10].

Stress was defined by Lazarus as a relationship between the person and the environment where the individual appraises the situation as personally significant and perceives it as imposing demands that exceed their coping resources [11]. Thus, stress is defined as "a transaction between the individual and the environment" [12]. Stress is an event that induces biochemical, physiological, psychological, and behavioral changes. Faced with the stressor, there is activation of the sympathetic system and release of catecholamines (adrenaline and noradrenaline), resulting in changes in the cardiovascular system, including an increase in heart rate and blood pressure [13].

Job stress or work-related stress or occupational stress is defined by the National Institute for Occupational Safety and Health (NIOSH) as harmful physical and emotional reactions that occur when the job demands do not match with workers' capabilities, resources, or needs [14]. The workplace has an important influence on the well-being and health of workers. Exposure to stressful working conditions (job stressors) can influence the health of workers [14, 15]. Under prolonged stress, adaptive capacities (physical and biological) remain limited and when stressors accumulate excessively, the individual feels progressively overwhelmed, which can be harmful and cause health problems for workers [16]. Health and social services, administration, education, the banking and insurance sector, transport, restaurants and hotels, and the police would be among the most affected sectors by job stress [17].

Two main reference models prevail in the literature to assess job stress: the Job Strain model developed by Karasek [18] and the Effort-Reward Imbalance model developed by Siegrist [19]. According to Karasek, "Job strain" is a situation combining high psychological demand with low decision latitude. There are several versions of this model. However, one of the most widely used is the 26-item version, which is defined by a psychological demand score below 21 and decision latitude below 71 [20]. Several studies have confirmed the predictive effects of these models on health, especially cardiovascular and mental health [21, 22]. Thus, several studies found an association between HTN and job stress, and stress is a risk factor for elevated blood pressure in both men and women in the workplace [10, 23]. Job stress has also been associated with uncontrolled blood pressure [24, 25].

Although many studies have investigated the association between job stress and HTN and it is still debated, most of these studies were carried out in Europe, North America, and Asia. In Sub-Saharan Africa, there are a very limited number of studies on the subject. This scoping review aims to explore current evidence of knowledge on HTN and job stress in that area.

## Methods

### Research design

A scoping review was conducted to identify the literature available on HTN and job stress in Sub-Saharan Africa. This review method was deemed most suitable for exploring the research carried out in this area and to identify knowledge gaps in the literature [26]. We conducted this scoping review according to Arksey and O'Malley methodology framework [27], and we followed a five-step approach, which included (i) establishing the research question, (ii) identifying relevant studies, (iii) selecting studies, (iv) charting the data, and (v) collating, summarizing, and reporting the results [27]. The PCC model (Population, Concepts, and Context) was used to construct the research question as proposed by Peter et al. including Population (workers), Concepts (hypertension and job stress), and Context (Sub-Saharan Africa) [28]. To provide complete and transparent reporting, we used the Preferred Reporting Items for Systematic Reviews and Meta-Analyses extension for Scoping Reviews (PRISMA-ScR) checklist [29].

### Identification of relevant studies

Three electronic databases (PubMed, Scopus, and ProQuest) were used on March 1, 2023, to identify publications with the assistance of a health sciences librarian. To identify relevant titles and abstracts, a systematic search was conducted across each database. A combination of keywords, search terms, and Medical Subject Headings using the AND and OR Boolean operators was employed in the search strategies. To restrict our search to Sub-Saharan Africa, we applied a filter based on country. Keywords included terms related to HTN [Hypertension, High blood pressure, Cardiovascular Risk Factor, Risk Factors for Cardiovascular Disease, Heart Disease Risk Factors, Risk Factors for Heart Disease, Risk Factors for Heart Diseases], job stress [Occupational stress, Job related Stress, Job Stress, Professional Stress, Work related Stress, Workplace Stress, stress at work, job strain, Psychosocial work environment, Psychosocial factor, Psychosocial risk factor, Effort-reward imbalance, Déséquilibre effort-récompense, Stress au travail, Stress professionnel] and to Sub-Saharan Africa, where all the countries in this region have been listed to apply the filter. The full electronic search strategy used for each of these databases is provided in the appendix (Additional file 1). Also, we conducted a manual search through the references of the included studies on Google Scholar to identify additional articles.

### Study selection

All the manuscripts identified with the keywords were imported into the referencing software EndNote to delete duplicates. In a second step, the articles were assessed using Rayyan software independently by two reviewers by title and abstract to check whether they met the inclusion criteria. Studies that fulfill the following criteria: (i) Peer-reviewed manuscripts published in journals without date restriction until March 2023, (ii) Studies with quantitative methodologies, (iii) Participants were workers or studies in the workplace, (iv) Studies that reported HTN or high blood pressure and job stress, and (v) Studies published in English or French, were included. In the third step, the full text of the selected studies was assessed independently by two reviewers to verify that they met the inclusion criteria and were retained for data extraction and analysis.

All disagreements about the inclusion or exclusion of articles were discussed during each of these steps. A consensus was reached based on the relevance of the arguments mentioned (noted), and a third researcher was involved as a referee when a consensus decision was not reached.

### Charting the data

A data charting form was developed to extract various variables through an iterative approach. Data extracted were imported into a Microsoft Excel spreadsheet. All disagreements were solved by consensus based on the arguments. A third researcher was involved as a referee when a consensus decision was not reached.

For each study, data extracted included the author's name, year of publication, country where the study was conducted, study design, sample size, age and range of participants, information related to the measurement of blood pressure and job stress, and main findings.

### Collating, summarizing, and reporting the results

An analysis was conducted on the selected articles through the extracted data. The findings were collated and presented in a consolidated table with the geographical distribution of the studies' locations. A narrative summary provides an overview of these data on the current state of the literature on HTN and job stress in Sub-Saharan Africa.

### Quality appraisal of included articles

To evaluate the quality of studies included in the review, we used the critical appraisal tools of the Joanna Briggs Institute (JBI) which assist in assessing the trustworthiness, relevance, and results of published papers [30]. A specific form in the Microsoft Excel spreadsheet with the tool's criteria for analytical cross-sectional studies and case–control studies format was used. Studies were classified into 3 categories according to their quality: low (< 50%), medium (50 to 74%), and high (⩾75%).

## Results

### Search results

**Fig. 1:**
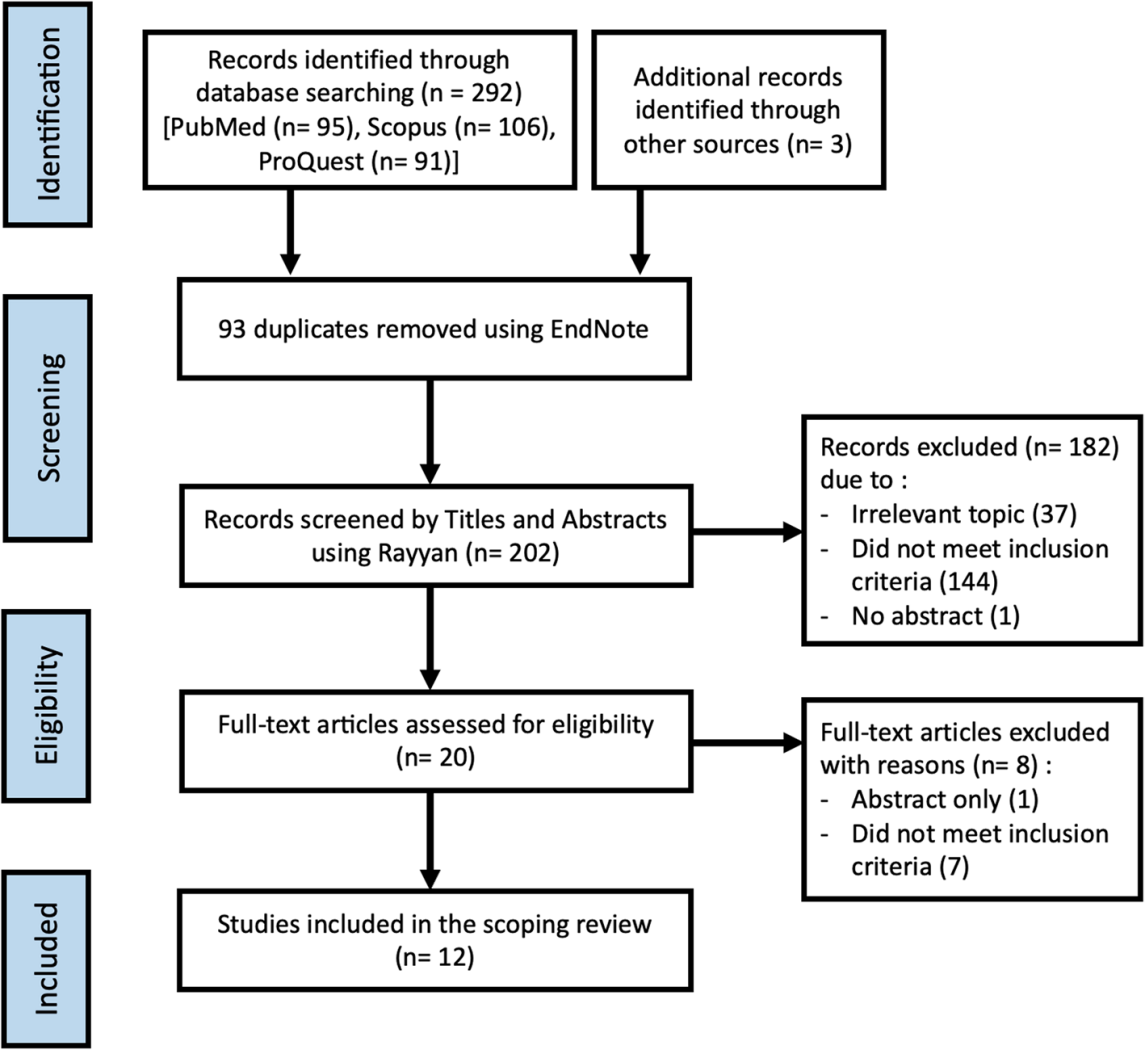
PRISMA Flow Diagram for inclusion process of articles in the review

A total of 295 articles were identified, mainly in the three electronic databases and only 3 additional records were manually found. Moreover, 93 duplicate articles were removed, and the remaining 202 were screened according to the inclusion criteria. After a review of the title and abstracts, 20 articles were included for the full-text review. Furthermore, 8 articles were excluded during the full-text screening regarding the inclusion criteria. Finally, 12 articles fulfilled the inclusion criteria and were included in the current scoping review (Fig. 1).

### Type of studies and objectives

**Fig. 2:**
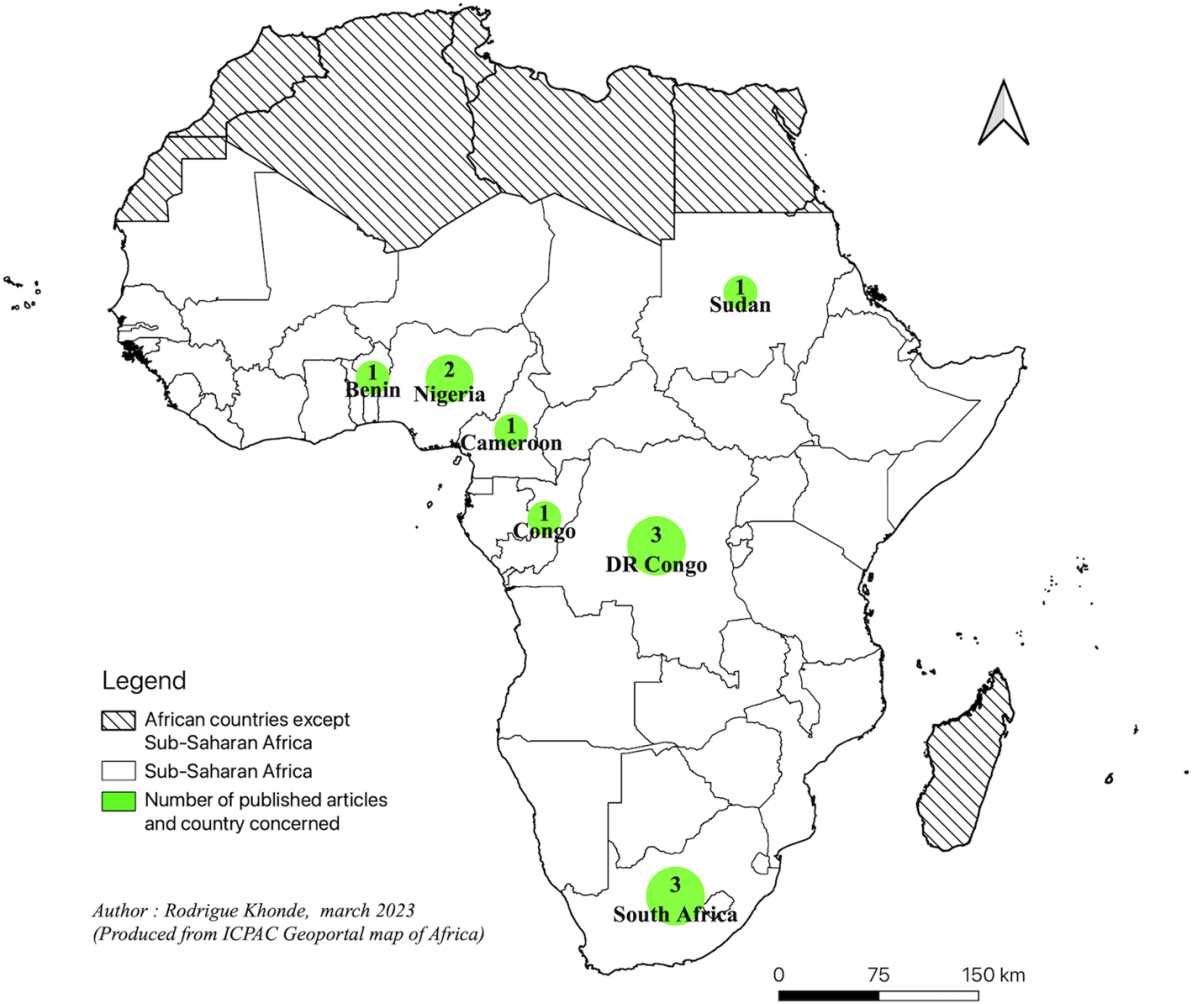
Hypertension and job stress: published articles in Sub-Saharan Africa

The 12 studies were published between 2003 and 2022. All studies employed quantitative measures. Of the studies included in the present scoping review, 8 were cross-sectional studies [31–38], and 3 case–control studies [39–41]. The design was not reported in a study, but the methodology followed was a cross-sectional study [42]. Regarding the geographical location of these studies, 3 were carried out in South Africa, 3 in the Democratic Republic of Congo, 2 in Nigeria, 1 in Benin, 1 in Cameroon, 1 in Congo, and 1 in Sudan. The geographical distribution of these studies is presented in the Fig. 2.

The objective of several studies was to determine the prevalence of HTN and associated factors in the workplace [33–35, 37]. Others investigated the relationship between job stress and HTN [39, 40] and another sought to determine the prevalence of HTN and other cardiovascular risk factors among workers [36].

### Quality appraisal

Of the 12 included articles, 3 were high-quality [32, 37, 38], 6 were medium-quality [31, 33–35, 39, 41], and 3 were low-quality [36, 40, 42] according to the Joanna Briggs Institute (JBI) critical appraisal tools used, providing a general overview of the quality of these studies (Additional file 2).

### Narrative analysis

The summary of study characteristics and findings of the included articles is presented in Table 1.

**Table 1:** Summary of study characteristics and findings

Ref	Author/Year/	Sample size	Study design	Age/range	Measurement		Prevalence		Summary of Findings
	Country			(years)	Blood pressure	Job stress	Hypertension	Job stress	
[42]	Promtussananon et al., 2003 South Africa	240	N.R	N.R 21–60	No measurement. Self-reported through a questionnaire	25-item perceived stress scale and 7-item sub-scale on stress management	N.R	22.6% (staff in rural hospitals), 19.7% (peri-urban), and 16.6% (urban)	1) HTN was inversely associated with stress management (r = -0.14, p < 0.05)
									2) Perceived stress was inversely positively correlated with HTN (r = 0.17, p < 0.05)
[36]	Gombet et al., 2007 Congo	145	Cross-sectional study	42.2 ± 9.1 22–61	Measurement of BP with a sphygmomanometer	No model. Reported job stress	34,5%	N.R	1) Job stress was reported in 70% of hypertensive participants
[31]	Peltzer et al., 2008 South Africa	21,307	Cross-sectional study	40 ± 8.1 18–69	No measurement. Self-reported through a questionnaire	A 6-item job stress index developed by modifying existing scales	N.R	N.R	1) Prevalence of stress-related illnesses was 15.6% for HTN
									2)HTN was found to be related to job stress among men (OR = 1.12 [1.06–1.20], p < 0.001) but not among women (OR = 1.03 [0.99–1.07], p = 0.21)
									3) Job stress and lack of job satisfaction were associated with most stress-related illnesses (HTN, tobacco, alcohol misuse…)
[38]	Owolabi et al., 2012 Nigeria	324	Cross-sectional descriptive study	41.1 ± 10.1 20–65	Measurement of BP with a sphygmomanometer	Model of Karasek	20.1%	26.2%	1) Male participants had higher job strain compared to female subjects (statistically significant, p < 0.05)
									2) The highest percentage of hypertensives was seen amongst the high job strain (42.4%) participants (3 times more hypertensives in this group compared with any other group)
									3) The association between high strain and increased prevalence of HTN was statistically significant (p < 0.05)
[33]	Sumaila et al., 2016 Nigeria	107	Cross-sectional study	32.3 ± 7.5 N.R	Measurement of BP with a sphygmomanometer	Stress Measuring Questionnaire (International Stress Management Association questionnaire)	26.2%	N.R	1) No significant correlation was found between HTN and job stress (r = -0.079, p = 0.133)
[32]	Aginsky et al., 2017 South Africa	603	Observational, retrospective chart audit of data from 6 companies’ health screening days	38.2 ± 9.7 N.R	Measurement of BP with a sphygmomanometer	Non-standard stress level assessment questionnaire	27.4%	N.R	1) White-collar workers (WCWs) were mostly male (90.5 vs. 42.5%; P < .01) and showed higher stress levels (P < 0.01) than the Blue-collar workers (BCWs)
									2) BCWs were significantly older, had higher blood pressure readings, and were more likely to develop HTN (OR: 1.72 [1.05–2.81] p = 0.03) than WCWs
[39]	Umba et al., 2020 DR Congo	84	Case–control study	N.R	Measurement of BP with a monitor	No model. Reported occupational stress	25%	N.R	1) 80.95% of nurses versus 19.05% of physicians had HTN
									2) Job stress (OR = 11.56 [2.45–110.82]) was associated with the occurrence of HTN
[40]	Nanga et al., 2020 Cameroon	637	Case–control study	52 ± 5.1 37–60	N.R	Model of Karasek	14.3%	N.R	1) Hypertensive workers had a family history of HTN in 38% of cases and HTN was significantly correlated with age (p < 0.001, OR = 16.95)
									2) In 63% of the cases, workers were in the "job strain" quadrant, of which 70% were operators (representing the largest percentage (66%) of hypertensive workers)
[37]	Panda et al., 2020 DR Congo	326	Analytical cross-sectional study	39.6 ± 0.6 20 –70	Measurement of BP with a monitor	Model of Karasek	32,5%	47.9%	1) 53.5% of workers with HTN did not know they had HTN
									2) Job stress was significantly associated with HTN (OR = 2.4 [1.5–4.4], p < 0.001)
[34]	Khaild et al., 2022 Sudan	98	Descriptive cross-sectional study	N.R	Measurement of BP	Not reported how job stress was assessed	45.9%	N.R	1) 40% of workers with HTN did not know they had HTN
									2) HTN was significantly associated with job stress (p < 0.005)
									3) Severe (OR = 3.4 [1.7–8.5], p = 0.004) and very severe work stress (OR = 6.5 [2.2–15.1], p < 0.001) were significantly associated with HTN
[35]	Adjobimey et al., 2022 Benin	86	Descriptive and analytical cross-sectional study	41.6 ± 9.8 23–70	Measurement of BP	Model of Karasek	19.8%	N.R	1) 70.6% of workers with HTN did not know they had HTN
									2) HTN was higher in workers under job stress (41.18%) compared to non-stressed workers (14.49%) (p = 0.032)
[41]	Kalumba et al., 2022 DR Congo	201	Nested case–control type	48,5 ± 11,1 29–79	Measurement of BP	Combined Karasek-Siegrist model	20%	40%	1) Self-measurement of BP noted an increase in BP ≥ 135/85 mmHg in 27% of teachers
									2) No statistically significant difference was found between job stress and increased BP

Data of age are mean ± standard deviation; N.R. indicates data “not reported” in the article

#### Study participants

The sample size of included studies ranged between 84 participants in the Democratic Republic of Congo [39] and 21,307 participants in South Africa [31]. The ages of the subjects ranged from 18 to 79 years. However, 4 of these studies did not report the age range [32–34, 39]. All the studies included both male and female participants.

#### Measurement of exposure

Job stress measurement was performed in 9 studies [31–33, 35, 37, 38, 40–42]. The most widely used stress assessment model is the Karasek model in 5 studies with the 26-item version. The “Job strain” was defined by a psychological demand of less than 21 and a decision latitude of less than 71 [35, 37, 38, 40, 41]. Although, only one study did not describe how stress was assessed following this model [41].

In addition to that exposure measurement, the measurement of blood pressure (BP) was done in 10 studies [32–41]. HTN was defined as SBP ⩾140 mmHg and by DBP ⩾90 mmHg. Nevertheless, 2 studies did not report how HTN was defined [33, 40]. Only 6 reported the measurement of both job stress exposure and blood pressure.

#### Activity sectors covered by the included studies

Four studies were carried out in the health sector [33, 38, 39, 42], 2 in the banking sector [34, 36], 2 in the education sector [31, 41], 2 in the industry sector [37, 40], 1 in the administration sector [35] and 1 multi-sector worker study among blue-collar workers (mining, manufacturing, and construction) and white-collar workers (banking, IT, and retail companies) [32].

#### Prevalence of hypertension and job stress

The prevalence of HTN ranged from 14.3% [40] to 45.9% [34]. A high proportion of hypertensive participants (35.4% to 70.6%) were unaware that they had HTN at the time of the study [33–35, 37]. A study highlighted that blood pressure was higher in males than in females [36]. The prevalence of job stress ranged from 16.6% [42] to 47.9% [37]. The stress level was related to occupation and among these most stressed subjects, we also had the highest number of hypertensives [32, 39, 40]. This stress level was also highlighted higher in males than in females [31, 38].

#### Association between hypertension and job stress

In total, 9 studies reported a relationship between HTN and job stress [31, 34–40, 42]. These studies noted that an increase in a job stress situation was associated with an increase in blood pressure. The perceived stress among workers was positively associated with HTN (*r* = 0.17, *p* < 0.05) [42]. Of the studies that reported an association between HTN and job stress, only 2 studies adjusted for other factors. Thus, after adjustment, job stress (OR = 2.4 [1.5–4.4], *p* < 0.001) was significantly associated with HTN in the first study [37], and in the second, that was statistically significant only in men (OR = 1.12 [1.06–1.20], *p* < 0.001) [31].

Other factors known as traditional risk factors of HTN have been reported as associated with HTN, such as age [33, 34, 37]; gender where males were more likely to be affected by HTN (OR = 1.12 [1.06–1.20]) [31], (OR = 2.2 [1.3—3.7]) [37]; heredity or family history of HTN (OR = 2.4 [1.3—4.7]) [37], also highlighted by Khaild et al*.* [34] and Adjobimey et al*.* [35]; overweight (OR = 2.9 [1.4—6.1]) or obesity (OR = 4.3 [1.9—9.8]) [37] that were also identified in other studies [33–35]; alcohol consumption (OR = 7.0 [2.36–20.70]) [39], reported as well in 2 studies [31, 36]; smoking [31, 34, 36, 39]; physical activity (OR = 2.3 [1.2—4.3]) [37], (*r *= -0.193, *p* = 0.001) [33], found also in other studies [34, 36].

#### Stress management and health promotion program in the workplace

None of the studies documented the availability of a workplace health promotion program, particularly concerning cardiovascular disease risk factors. However, a study that assessed stress management among healthcare workers in hospitals noted that HTN was inversely associated with stress management (*r* = -0.14, *p* < 0.05) [42]. Nevertheless, this evaluation did not mention a specific job stress management program implemented in these hospitals. The authors suggest that effective lifestyle and health promotion programs are needed to reduce stress and health risks for healthcare workers.

## Discussion

The present paper synthesizes the existing knowledge about HTN and job stress in Sub-Saharan Africa. Globally, there are few publications on this topic. The quality appraisal (JBI critical appraisal tools) illustrated that half of the studies identified and included are of medium quality. The model most commonly used in these studies to assess exposure (job stress) was Karasek's model. The values used for the definition of the "Job strain" situation of Karasek’s model, associating high psychological demand with low decision latitude, are comparable with those reported in studies carried out in other regions of the world for the 26 items version, for which the psychometric properties of the French version have been validated [20]. This situation is reported to be associated with a high risk of cardiovascular disease, especially when the worker has low social support [43].

The studies were conducted on sectors of activity described in the literature where workers are most exposed to job stress [17, 44]. Overall, most studies reported an association between HTN and job stress among workers in the workplace. However, only one study also mentioned the relationship between stress management and HTN. HTN has also been reported to be associated with job stress in several studies, including the meta-analysis where, for cross-sectional studies, a single exposure to “job strain” was associated with SBP (3.43 mm Hg [2.02, 4.84], *p* < 0.001, *I*^*2*^ = 62.3) and DBP (2.07 mm Hg [1.17, 2.97], *p* < 0.001, *I*^*2*^ = 42.3) at work [23]. Nevertheless, this association remains controversial. Another meta-analysis did not find an effect of association between job stress and HTN [45].

The review showed a high prevalence of HTN among participants, including a significant proportion who did not know they had HTN at the time of the study. These results are consistent with a cohort study conducted across four countries in Sub-Saharan Africa aimed to determine the prevalence of pre-HTN, HTN, and associated factors, assessing of BP data collected revealed that only 50% of participants with HTN were aware of their elevated blood pressure condition [46]. These results support the view that HTN is a health problem among workers in Sub-Saharan Africa that deserves to be considered in the workplace. However, the failure to control for certain confounding factors in most of these studies may bring into doubt the significant associations found in some studies and/or the lack of association found in others.

The design of the studies carried out does not allow a causal link to be established between HTN and job stress. No longitudinal studies of a cohort of workers with repeated measures or randomized controlled trials with a follow-up period have been conducted in the workplace, compared to studies in other parts of the world [23, 47]. Given the increasing rates of HTN in Sub-Saharan Africa, the existence of a prevention or health promotion program related to cardiovascular disease or cardiovascular risk factors was not reported in these studies. Workers represent half of the world's population, and their health is critical to productivity and economic development. Their health is determined by occupational hazards, social and individual factors, and by access to health services. A focus should be placed on the prevention of occupational hazards and the promotion of health in the workplace [48]. The fact that no health promotion programs are mentioned as being implemented in the workplace in Sub-Saharan Africa raises concerns about the real consideration of the burden of cardiovascular disease in countries of this region. However, the implementation of lifestyle or stress management interventions in the workplace has been reported elsewhere to affect reducing blood pressure in workers [49–51]. This is the case of a prospective cohort study carried out in Malaysia among employees in a university, where a workplace health promotion program was conducted and reported a decrease of 2.36 mmHg among SBP in the HTN subgroup (*p* < 0.0001) and a significant improvement in SBP among the participants who were at risk of HTN (-0.75 mmHg, *p* < 0.001) [50].

### Limitations

Our scoping review has some limitations. Although keywords were used in electronic search databases and a manual search completed this selection step, it is difficult to identify all the articles published on this topic and achieve exhaustiveness, which could constitute a selection bias. In addition, the exploratory study does not allow a quantitative assessment of the included studies. Given the design of the studies included in our exploratory review (cross-sectional and case–control studies), the available data on the subject are limited and do not allow us to establish a causal link between HTN and job stress.

## Conclusion

Our scoping review of the available literature found an increasing prevalence of HTN in the workplace, with a high proportion of workers who are unaware that they have HTN. Studies have shown also that job stress is associated with HTN. Our analysis of the published literature also found gaps in terms of existing health programs or strategies for stress management or lifestyle promotion in the workplace. Given the increasing prevalence of HTN, there is a need for prevention and health promotion policies in the workplace to be implemented in Sub-Saharan Africa about the control of cardiovascular disease to achieve sustainable development goal 3 about non-communicable diseases.

### Supplementary Information

**Additional file 1. **Search strategy

**Additional file 2. **Quality appraisal of included articles

## Data Availability

All data generated or analyzed during this study are included in this published article [and its supplementary information files].

## Abbreviations

HTN: Hypertension
SBP: Systolic blood pressure
DBP: Diastolic blood pressure
JBI: Joanna Briggs Institute
N.R.: Not reported
