# Supplementary material for: Job stress, a source of hypertension among workers in Sub-Saharan Africa: a scoping review
Source: BMC Public Health. 2023 Nov 23;23:2316. doi: 10.1186/s12889-023-17248-5 (PMC10666436; doi:10.1186/s12889-023-17248-5)
Supplement: Supplementary file 1 — Additional file 1. Search strategy [file 12889_2023_17248_MOESM1_ESM.docx]

**ADDITIONAL FILE 1: SEARCH STRATEGY**

PCC model with

- Population (workers),
- Concepts (hypertension and job stress) and,
- Context (sub-Saharan Africa)

| Concepts/ Keywords | Synonyms |
| --- | --- |
| Worker | Worker |
| Job Stress | Occupational stress; Job related Stress; Job Stress; Professional Stress; Work related Stress; Workplace Stress; Workplace; Work place; stress at work; job strain; Psychosocial work environment; Psychosocial factor; Psychosocial risk factor; Effort-reward imbalance; Déséquilibre effort-récompense; Stress au travail; Stress professionnel |
| Hypertension | Hypertension; High blood pressure; Cardiovascular Risk Factor; Risk Factors for Cardiovascular Disease; Heart Disease Risk Factors; Risk Factors for Heart Disease; Risk Factors for Heart Diseases |
| Sub-Saharan Africa | Africa South of the Sahara, Sub-Saharan Africa; Subsaharan Africa; ANGOLA; BENIN; BOTSWANA; BURKINA FASO; CABO VERDE; CAMEROON; CENTRAL AFRICAN REPUBLIC; CHAD; CONGO; COTE D'IVOIRE; DEMOCRATIC REPUBLIC OF THE CONGO; DJIBOUTI; EQUATORIAL GUINEA; ERITREA; ESWATINI; SWAZILAND; ETHIOPIA; GABON; GAMBIA; GHANA; GUINEA; GUINEA-BISSAU; KENYA; LESOTHO; LIBERIA; MALAWI; MALI; MAURITANIA; MOZAMBIQUE; NAMIBIA; NIGER; NIGERIA; RWANDA; SAO TOME AND PRINCIPE; SENEGAL; SIERRA LEONE; SOMALIA; SOUTH AFRICA; SOUTH SUDAN; SUDAN; TANZANIA; TOGO; UGANDA; ZAMBIA; and ZIMBABWE |

**PubMed**

**(95 ARTICLES)**

((("Occupational Stress"[Mesh] OR "Occupational stress"[Text Word] OR "Job related Stress"[Text Word] OR "Job Stress"[Text Word] OR "Professional Stress"[Text Word] OR "Work related Stress"[Text Word] OR "Workplace Stress"[Text Word] OR "Work place Stress"[Text Word] OR "Work Stress"[Text Word] OR "Stress at work"[Text Word] OR "Job strain"[Text Word] OR "Effort-reward imbalance"[Text Word]) OR ("Workplace"[Text Word] OR "Work place"[Text Word] OR "Psychosocial work environment"[Text Word] OR "Psychosocial factor"[Text Word] OR "Psychosocial risk factor"[Text Word] OR "Worker"[Text Word])) AND (("Hypertension"[Mesh] OR Hypertension[Text Word] OR "High blood pressure"[Text Word] OR "Cardiovascular Risk Factor"[Text Word] OR "Risk Factors for Cardiovascular Disease"[Text Word] OR "Heart Disease Risk Factors"[Text Word] OR "Risk Factors for Heart Disease"[Text Word] OR "Risk Factors for Cardiovascular Diseases"[Text Word] OR "Heart Diseases Risk Factors"[Text Word] OR "Risk Factors for Heart Diseases"[Text Word] OR "Heart Disease Risk Factors"[Mesh]))) AND (("Africa South of the Sahara"[Mesh] OR "Africa South of the Sahara"[Text Word] OR "Sub-Saharan Africa"[Text Word] OR "Subsaharan Africa"[Text Word] OR ANGOLA[Text Word] OR BENIN[Text Word] OR BOTSWANA[Text Word] OR "BURKINA FASO"[Text Word] OR "CABO VERDE"[Text Word] OR CAMEROON[Text Word] OR "CENTRAL AFRICAN REPUBLIC"[Text Word] OR CHAD[Text Word] OR CONGO[Text Word] OR "COTE D'IVOIRE"[Text Word] OR "IVORY COAST"[Text Word] OR "DEMOCRATIC REPUBLIC OF THE CONGO"[Text Word] OR DJIBOUTI[Text Word] OR "EQUATORIAL GUINEA"[Text Word] OR ERITREA[Text Word] OR ESWATINI[Text Word] OR SWAZILAND[Text Word] OR ETHIOPIA[Text Word] OR GABON[Text Word] OR GAMBIA[Text Word] OR GHANA[Text Word] OR GUINEA[Text Word] OR "GUINEA-BISSAU"[Text Word] OR KENYA[Text Word] OR LESOTHO[Text Word] OR LIBERIA[Text Word] OR MALAWI[Text Word] OR MALI[Text Word] OR MAURITANIA[Text Word] OR MOZAMBIQUE[Text Word] OR NAMIBIA[Text Word] OR NIGER[Text Word] OR NIGERIA[Text Word] OR RWANDA[Text Word] OR "SAO TOME AND PRINCIPE"[Text Word] OR SENEGAL[Text Word] OR "SIERRA LEONE"[Text Word] OR SOMALIA[Text Word] OR "SOUTH AFRICA"[Text Word] OR "SOUTH SUDAN"[Text Word] OR SUDAN[Text Word] OR TANZANIA[Text Word] OR TOGO[Text Word] OR UGANDA[Text Word] OR ZAMBIA[Text Word] OR ZIMBABWE[Text Word]))

**Scopus**

**(106 ARTICLES)**

(TITLE-ABS-KEY ( "Occupational stress" OR "Job related Stress" OR "Job Stress" OR "Professional Stress" OR "Work related Stress" OR "Workplace Stress" OR "Work place Stress" OR "Work Stress" OR "Stress at work" OR "Job strain" OR "Effort-reward imbalance" OR " Déséquilibre effort-récompense" OR "Stress au travail" OR "Stress professionnel" OR "Psychosocial work environment" OR "Psychosocial factor" OR "Psychosocial risk factor" OR "Workplace") OR (TITLE-ABS-KEY ("Workers") AND TITLE-ABS-KEY ( "Occupational stress" OR "Job related Stress" OR "Job Stress" OR "Professional Stress" OR "Work related Stress" OR "Workplace Stress" OR "Work place Stress" OR "Work Stress" OR "Stress at work" OR "Job strain" OR "Effort-reward imbalance" OR " Déséquilibre effort-récompense" OR "Stress au travail" OR "Stress professionnel" OR "Psychosocial work environment" OR "Psychosocial factor" OR "Psychosocial risk factor" OR "Workplace"))) AND TITLE-ABS-KEY ( "Hypertension" OR "High blood pressure" OR "Cardiovascular Risk Factor" OR "Risk Factors for Cardiovascular Disease" OR "Heart Disease Risk Factors" OR "Risk Factors for Heart Disease" OR "Risk Factors for Cardiovascular Diseases" OR "Heart Diseases Risk Factors" OR "Risk Factors for Heart Diseases" ) AND TITLE-ABS-KEY ( "AFRICA SOUTH OF THE SAHARA" OR "SUB-SAHARAN AFRICA" OR "SUBSAHARAN AFRICA" OR "ANGOLA" OR "BENIN" OR "BOTSWANA" OR "BURKINA FASO" OR "CABO VERDE" OR "CAP-VERT" OR "CAMEROON" OR "CAMEROUN" OR "CENTRAL AFRICAN REPUBLIC" OR "REPUBLIQUE CENTRAFRICAINE" OR "CHAD" OR "TCHAD" OR "CONGO" OR "COTE D'IVOIRE" OR "IVORY COAST" OR "DEMOCRATIC REPUBLIC OF THE CONGO" OR "REPUBLIQUE DEMOCRATIQUE DU CONGO" OR "DJIBOUTI" OR "EQUATORIAL GUINEA" OR "GUINEE EQUATORIALE" OR "ERITREA" OR "ERYTHREE" OR "ESWATINI" OR "SWAZILAND" OR "ETHIOPIA" OR "ETHIOPIE" OR "GABON" OR "GAMBIA" OR "GAMBIE" OR "GHANA" OR "GUINEA" OR "GUINEA-BISSAU" OR "GUINEE-BISSAU" OR "KENYA" OR "LESOTHO" OR "LIBERIA" OR "MALAWI" OR "MALI" OR "MAURITANIA" OR "MAURITANIE" OR "MOZAMBIQUE" OR "NAMIBIA" OR "NAMIBIE" OR "NIGER" OR "NIGERIA" OR "RWANDA" OR "SAO TOME AND PRINCIPE" OR "SAO TOME-ET-PRINCIPE" OR "SENEGAL" OR "SIERRA LEONE" OR "SOMALIA" OR "SOUTH AFRICA" OR "AFRIQUE DU SUD" OR "SOUTH SUDAN" OR "SOUDAN DU SUD" OR "SUDAN" OR "SOUDAN" OR "TANZANIA" OR "TANZANIE" OR "TOGO" OR "UGANDA" OR "OUGANDA" OR "ZAMBIA" OR "ZAMBIE" OR "ZIMBABWE" )

**ProQuest**

**(91 ARTICLES)**

noft(((("Occupational stress" OR "Job related Stress" OR "Job Stress" OR "Professional Stress" OR "Work related Stress" OR "Workplace Stress" OR "Work place Stress" OR "Work Stress" OR "Stress at work" OR "Job strain" OR "Effort-reward imbalance" OR " Déséquilibre effort-récompense" OR "Stress au travail" OR "Stress professionnel") OR ("Psychosocial work environment" OR "Psychosocial factor" OR "Psychosocial risk factor" OR "Workplace" OR "Worker")) AND ("Hypertension" OR "High blood pressure" OR "Cardiovascular Risk Factor" OR "Risk Factors for Cardiovascular Disease" OR "Heart Disease Risk Factors" OR "Risk Factors for Heart Disease" OR "Risk Factors for Cardiovascular Diseases" OR "Heart Diseases Risk Factors" OR "Risk Factors for Heart Diseases")) AND ("AFRICA SOUTH OF THE SAHARA" OR "SUB-SAHARAN AFRICA" OR "SUBSAHARAN AFRICA" OR "ANGOLA" OR "BENIN" OR "BOTSWANA" OR "BURKINA FASO" OR "CABO VERDE" OR "CAP-VERT" OR "CAMEROON" OR "CAMEROUN" OR "CENTRAL AFRICAN REPUBLIC" OR "REPUBLIQUE CENTRAFRICAINE" OR "CHAD" OR "TCHAD" OR "CONGO" OR "COTE D'IVOIRE" OR "IVORY COAST" OR "DEMOCRATIC REPUBLIC OF THE CONGO" OR "REPUBLIQUE DEMOCRATIQUE DU CONGO" OR "DJIBOUTI" OR "EQUATORIAL GUINEA" OR "GUINEE EQUATORIALE" OR "ERITREA" OR "ERYTHREE" OR "ESWATINI" OR "SWAZILAND" OR "ETHIOPIA" OR "ETHIOPIE" OR "GABON" OR "GAMBIA" OR "GAMBIE" OR "GHANA" OR "GUINEA" OR "GUINEA-BISSAU" OR "GUINEE-BISSAU" OR "KENYA" OR "LESOTHO" OR "LIBERIA" OR "MALAWI" OR "MALI" OR "MAURITANIA" OR "MAURITANIE" OR "MOZAMBIQUE" OR "NAMIBIA" OR "NAMIBIE" OR "NIGER" OR "NIGERIA" OR "RWANDA" OR "SAO TOME AND PRINCIPE" OR "SAO TOME-ET-PRINCIPE" OR "SENEGAL" OR "SIERRA LEONE" OR "SOMALIA" OR "SOUTH AFRICA" OR "AFRIQUE DU SUD" OR "SOUTH SUDAN" OR "SOUDAN DU SUD" OR "SUDAN" OR "SOUDAN" OR "TANZANIA" OR "TANZANIE" OR "TOGO" OR "UGANDA" OR "OUGANDA" OR "ZAMBIA" OR "ZAMBIE" OR "ZIMBABWE"))
