# Supplementary material for: Job stress, a source of hypertension among workers in Sub-Saharan Africa: a scoping review
Source: BMC Public Health. 2023 Nov 23;23:2316. doi: 10.1186/s12889-023-17248-5 (PMC10666436; doi:10.1186/s12889-023-17248-5)
Supplement: Supplementary file 2 — Additional file 2. Quality appraisal of included articles [file 12889_2023_17248_MOESM2_ESM.docx]

**Additional file 2: Quality appraisal of included articles**

| **RATING** | **YES=2** | **UNCLEAR=1** | **NO=0** | **NA=* (NA will not be considered in the calculation of the sum)** | |  |  |
| --- | --- | --- | --- | --- | --- | --- | --- |
|  |  |  |  |  |  | |  |
| **RATING OF QUALITY OF ARTICLES** | **≥75%=HIGH** | **50-74%= MEDIUM** | **<50%= LOW** |  |  | |  |

**Joanna Briggs Institute (JBI) CRITICAL APPRAISAL CHECKLIST FOR ANALYTICAL CROSS SECTIONAL STUDIES**

| **N°** | **Article title** | **Study ID** | **1.Were the criteria for inclusion in the sample clearly defined?** | **2.Were the study subjects and the setting described in detail?** | **3.Was the exposure measured in a valid and reliable way?** | **4.Were objective, standard criteria used for measurement of the condition?** | **5.Were confounding factors identified?** | **6.Were strategies to deal with confounding factors stated?** | **7.Were the outcomes measured in a valid and reliable way?** | **8.Was appropriate statistical analysis used?** | **Total score** | **Percentage of score** | **Quality rating of the article** |
| --- | --- | --- | --- | --- | --- | --- | --- | --- | --- | --- | --- | --- | --- |
| 1 | Health behaviour, perceived stress and stress management among hospital staff in the Limpopo Province, South Africa | Promtussananon et al., 2003 | 2 | 1 | 1 | 0 | 0 | 0 | 1 | 1 | 6 | 38 | LOW |
| 2 | Hypertension artérielle et autres facteurs de risque cardiovasculaires en milieu professionnel brazzavillois | Gombet et al., 207 | 1 | 1 | 0 | 2 | 0 | 0 | 1 | 1 | 6 | 38 | LOW |
| 3 | Job stress, job satisfaction and stress-related illnesses among South African educators | Peltzer et al., 2008 | 2 | 1 | 1 | 0 | 1 | 2 | 2 | 2 | 11 | 69 | MEDIUM |
| 4 | Work-related stress perception and hypertension amongst health workers of a mission hospital in Oyo State, south-western Nigeria | Owolabi et al., 2012 | 2 | 2 | 2 | 2 | 0 | 0 | 2 | 2 | 12 | 75 | HIGH |
| 5 | Prevalence of Undiagnosed Hypertension and its risk factors among health care workers of some selected hospitals in Dutse, Jigawa State, North western Nigeria | Sumaila et al., 2016 | 2 | 2 | 2 | 1 | 0 | 0 | 2 | 2 | 11 | 69 | MEDIUM |
| 6 | Cardiovascular disease risk profile and readiness to change in blue- and white-collar workers | Aginsky et al., 2017 | 2 | 1 | 1 | 2 | 1 | 2 | 2 | 2 | 13 | 81 | HIGH |
| 7 | Prevalence of hypertension at workplace and their risk factors in the Democratic Republic of Congo: Case of Kisangani's textile industry | Panda et al., 2020 | 2 | 2 | 2 | 2 | 0 | 2 | 2 | 2 | 14 | 88 | HIGH |
| 8 | Hypertension and Associated Risk Factors Among the Sudanese Banking Sector in River Nile State: A Descriptive Cross-Sectional Study | Khaild et al., 2022 | 2 | 1 | 0 | 2 | 0 | 0 | 2 | 2 | 9 | 56 | MEDIUM |
| 9 | Hypertension and Work Stress among City Hall Workers, Bohicon, Benin | Adjobimey et al., 2022 | 1 | 1 | 2 | 2 | 0 | 0 | 2 | 1 | 9 | 56 | MEDIUM |

| **RATING** | **YES=2** | **UNCLEAR=1** | **NO=0** | **NA=* (NA will not be considered in the calculation of the sum)** | |  |  |
| --- | --- | --- | --- | --- | --- | --- | --- |
|  |  |  |  |  |  | |  |
| **RATING OF QUALITY OF ARTICLES** | **≥75%=HIGH** | **50-74%= MEDIUM** | **<50%= LOW** |  |  | |  |

**Joanna Briggs Institute (JBI) CRITICAL APPRAISAL CHECKLIST FOR CASE CONTROL STUDIES**

| **N°** | **Article title** | **Study ID** | **1.Were the groups comparable other than the presence of disease in cases or the absence of disease in controls?** | **2.Were cases and controls matched appropriately?** | **3.Were the same criteria used for identification of cases and controls?** | **4.Was exposure measured in a standard, valid and reliable way?** | **5.Was exposure measured in the same way for cases and controls?** | **6.Were confounding factors identified?** | **7.Were strategies to deal with confounding factors stated?** | **8.Were outcomes assessed in a standard, valid and reliable way for cases and controls?** | **9.Was the exposure period of interest long enough to be meaningful?** | **10.Was appropriate statistical analysis used?** | **Total score** | **Percentage of score** | **Quality rating of the article** |
| --- | --- | --- | --- | --- | --- | --- | --- | --- | --- | --- | --- | --- | --- | --- | --- |
| 10 | Facteurs de risque associés à l’hypertension artérielle (HTA) chez les personnels soignants. Cas de l’Hôpital Gécamines Sud de Lubumbashi | Umba et al., 2020 | 2 | 1 | 2 | 0 | 1 | 0 | 0 | 2 | * | 1 | 9 | 50 | MEDIUM |
| 11 | High Blood Pressure from ALUCAM Group's Workers: the Impact of the Actvity | Nanga et al., 2020 | 2 | 0 | 1 | 2 | 1 | 0 | 0 | 1 | * | 1 | 8 | 44 | LOW |
| 12 | Stress professionnel et profil de la pression artérielle des enseignants du secteur primaire et secondaire de la ville de Lubumbashi en République Démocratique du Congo: Du 10 Janvier 2021 au 10 janvier 2022 | Kalumba et al., 2022 | 2 | 0 | 2 | 2 | 2 | 0 | 0 | 1 | * | 1 | 10 | 56 | MEDIUM |
